# Supplementary material for: Meta-analysis shows positive effects of plant diversity on microbial biomass and respiration
Source: Nat Commun. 2019 Mar 22;10:1332. doi: 10.1038/s41467-019-09258-y (PMC6430801; doi:10.1038/s41467-019-09258-y)
Supplement: Supplementary file 1 — Supplementary Information [file 41467_2019_9258_MOESM1_ESM.pdf]

Supplementary Information for

Meta-analysis shows positive effects of plant diversity on microbial biomass and respiration

Chen Chen[1], Han Y. H. Chen[1,2,*], Xinli Chen[1], Zhiqun Huang[2,3,*]

[1]Faculty of Natural Resources Management, Lakehead University, 955 Oliver Road, Thunder Bay, Ontario P7B 5E1, Canada

[2]Key Laboratory for Humid Subtropical Eco-geographical Processes of the Ministry of Education, Fujian Normal University, Fuzhou, 350007, China

[3]Institute of Geography, Fujian Normal University, Fuzhou, 350007, China

[*]Correspondence authors:

1. Han Y. H. Chen. Email: hchen1@lakeheadu.ca, Phone: +1 (807) 343-8342;

2. Zhiqun Huang. Email: zhiqunhuang@fjnu.edu.cn, Phone: +86 (591) 83434802.

Supplementary Figures

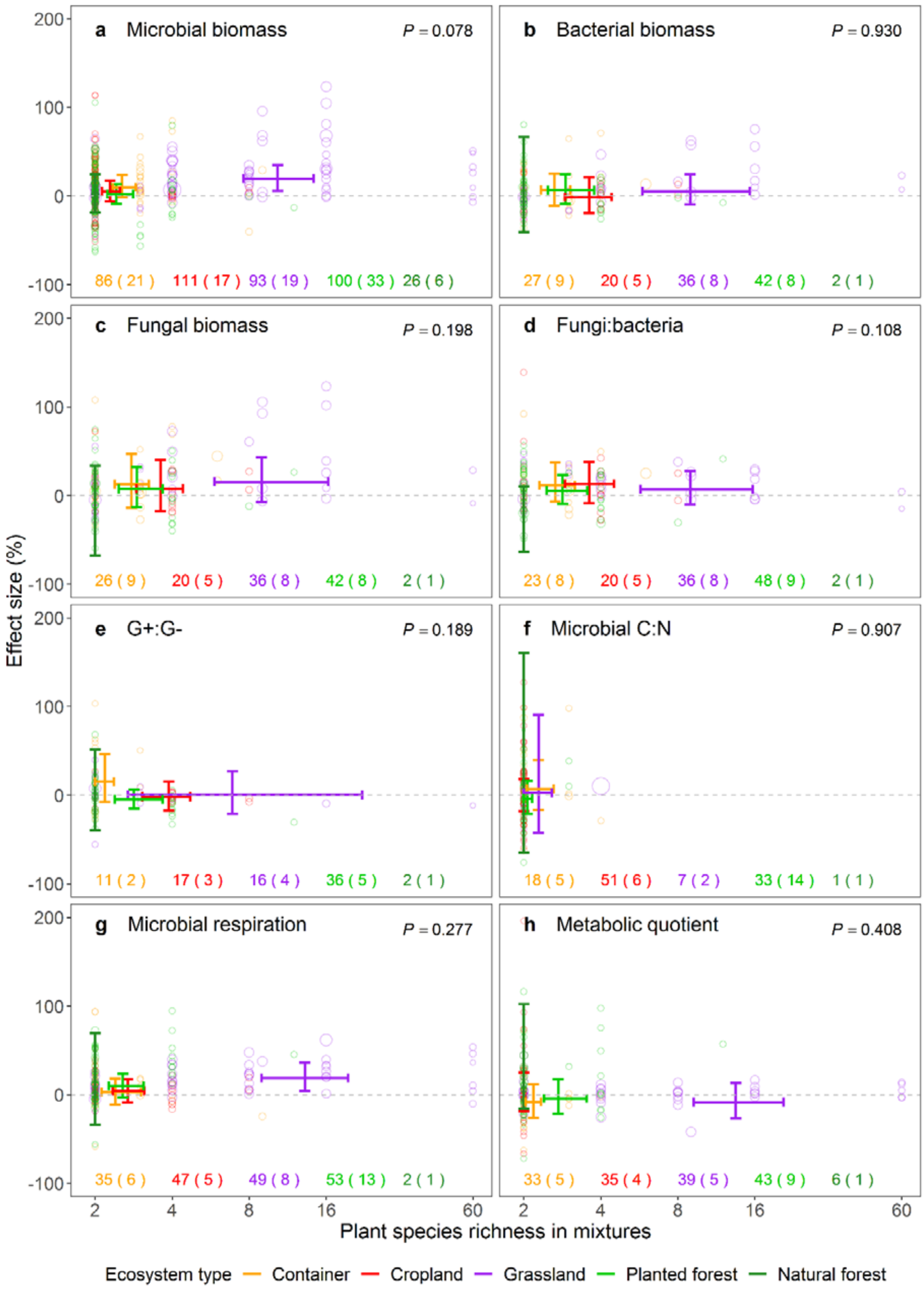

Supplementary Figure 1. Comparison of soil microbial attributes in plant mixtures versus monocultures and the species richness in mixtures among the five ecosystem types. Individual observations with point sizes corresponding to their weights (*Wr*, see Methods) are plotted by ecosystem type. **a** Microbial biomass. **b** bacterial biomass. **c** Fungal biomass. **d** Fungi:bacteria ratio. **e** gram-positive (G+) to gram-negative (G-) bacteria ratio. **f** microbial C:N ratio. **g** microbial respiration. **h** Metabolic quotient. Means and vertical and horizontal error bars represent means and 95% bootstrapped confidence intervals for the effect size (%) of plant mixtures and the species richness in mixtures, respectively. For each ecosystem type, the number of observations is shown without parentheses with the number of studies in parentheses. *P* value, derived from the linear mixed model with the ecosystem type as the fixed effect and study as the random effect, represents the significance of the difference in the effect sizes among ecosystem types. The figure shows that despite significant differences in the species richness in mixtures among ecosystem types (as indicated by non-overlapping CIs), the effect sizes did not significantly differ for any of the studied soil microbial attributes.

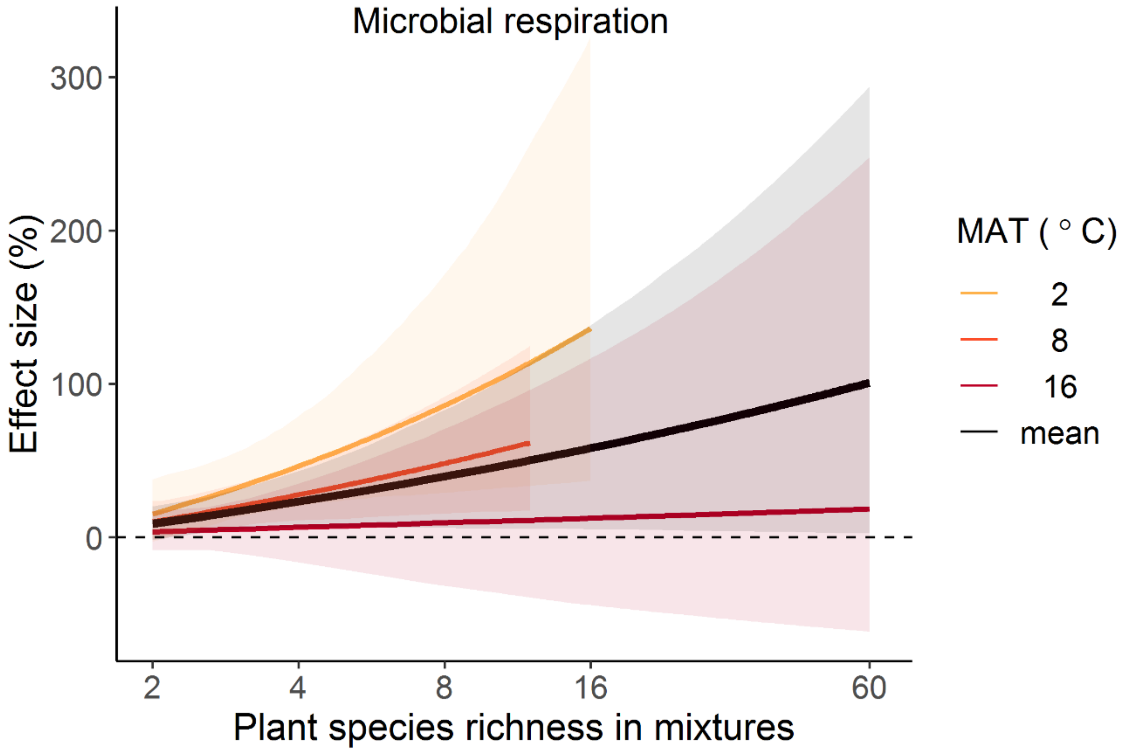

Supplementary Figure 2. Comparison of microbial respiration in relation to the species richness in mixtures and mean annual temperature (MAT). Lines are fitted MAT-dependent regressions and their bootstrapped 95% confidence intervals are shaded. Yellow, red, dark red and black lines indicate MAT at 2, 8 and 16 °C and average MAT across all observations under natural climate.

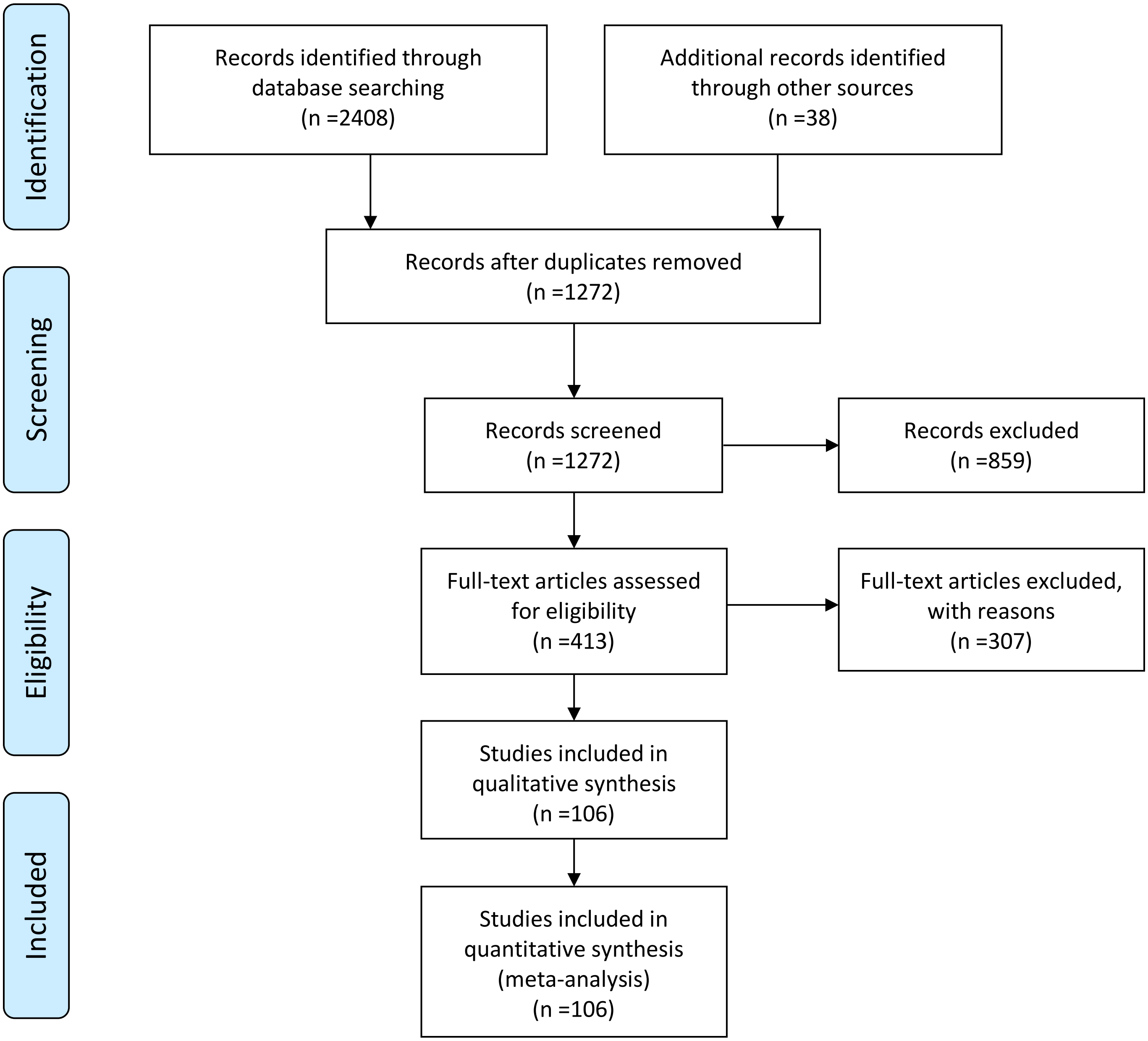

Supplementary Figure 3. PRISMA diagram showing the process of locating studies included in this meta-analysis.

Supplementary Tables

Supplementary Table 1. List of 106 papers used in the meta-analysis.

Data source	Reference number (from main text reference list)
Alvarez, G., Chaussod, R., Loiseau, P. & Delpy, R. Soil indicators of C and N transformations under pure and mixed grass-clover swards. *Eur. J. Agron.* **9**, 157-172 (1998).	42
Bagherzadeh, A., Brumme, R. & Beese, F. Temperature dependence of nitrogen mineralization and microbial status in O_H horizon of a temperate forest ecosystem. *J. For. Res.* **19**, 37-43 (2008).	43
Berger, T. W., Inselsbacher, E. & Zechmeister-Boltenstern, S. Carbon dioxide emissions of soils under pure and mixed stands of beech and spruce, affected by decomposing foliage litter mixtures. *Soil Biol. Biochem.* **42**, 986-997 (2010).	44
Bini, D., dos Santos, C. A., Bouillet, J. P., Goncalves, J. L. D. & Cardoso, E. *Eucalyptus grandis* and *Acacia mangium* in monoculture and intercropped plantations: Evolution of soil and litter microbial and chemical attributes during early stages of plant development. *Appl. Soil Ecol.* **63**, 57-66 (2013).	45
Borken, W. & Beese, F. Soil respiration in pure and mixed stands of European beech and Norway spruce following removal of organic horizons. *Can. J. For. Res.* **35**, 2756-2764 (2005).	46
Cesarz, S. *et al.* Roots from beech (*Fagus sylvatica* L.) and ash (*Fraxinus excelsior* L.) differentially affect soil microorganisms and carbon dynamics. *Soil Biol. Biochem.* **61**, 23-32 (2013).	47
Chen, J. & Yang, N. Effects of five plantations on soil properties in subtropical red soil hilly region (in Chinese with English abstract). *J. Northw. A&F Univ. (Natural Science Edition)* **41**, 167-173 (2013).	48
Chen, M. M. *et al.* Effects of soil moisture and plant interactions on the soil microbial community structure. *Eur. J. Soil Biol.* **43**, 31-38 (2007).	50
Chen, M. M., Chen, B. D. & Marschner, P. Plant growth and soil microbial community structure of legumes and grasses grown in monoculture or mixture. *J. Environ. Sci. (China)* **20**, 1231-1237 (2008).	49
Chodak, M. & Niklinska, M. Effect of texture and tree species on microbial properties of mine soils. *Appl. Soil Ecol.* **46**, 268-275 (2010).	51
Chodak, M. & Niklinska, M. The effect of different tree species on the chemical and microbial properties of reclaimed mine soils. *Biol. Fertility Soils* **46**, 555-566 (2010).	52
Chung, H., Zak, D. R., Reich, P. B. & Ellsworth, D. S. Plant species richness, elevated CO_2, and atmospheric nitrogen deposition alter soil microbial community composition and function. *Global Change Biol.* **13**, 980-989 (2007).	21

Cong, W.-F. & Eriksen, J. Forbs differentially affect soil microbial community composition and functions in unfertilized ryegrass-red clover leys. *Soil Biol. Biochem.* **121**, 87-94 (2018). 53

Coser, T. R. *et al.* Soil microbiological properties and available nitrogen for corn in monoculture and intercropped with forage. *Pesqui. Agrop. Bras.* **51**, 1660-1667 (2016). 54

De Deyn, G. B., Quirk, H. & Bardgett, R. D. Plant species richness, identity and productivity differentially influence key groups of microbes in grassland soils of contrasting fertility. *Biol. Lett.* **7**, 75-78 (2011). 55

Diaz-Pines, E. *et al.* Effects of tree species composition on the CO_2 and N_2O efflux of a Mediterranean mountain forest soil. *Plant Soil* **384**, 243-257 (2014). 56

Dijkstra, F. A., Hobbie, S. E., Reich, P. B. & Knops, J. M. H. Divergent effects of elevated CO_2, N fertilization, and plant diversity on soil C and N dynamics in a grassland field experiment. *Plant Soil* **272**, 41-52 (2005). 57

Dong, M. *et al.* Soil microbial biomass C, N and diversity characteristics in pure and mixed forest of *Pinus* and *Cinnamomun* (in Chinese with English abstract). *J. Centr. South Univ. For. & Techn.* **37**, 146-153 (2017). 58

Drissner, D., Blum, H., Tscherko, D. & Kandeler, E. Nine years of enriched CO_2 changes the function and structural diversity of soil microorganisms in a grassland. *Eur. J. Soil Sci.* **58**, 260-269 (2007). 59

Du, C. *Effects of potato/maize intercropping on soil and crops (in Chinese with English abstract).* Master's thesis, Ningxia University, (2017). 60

Duo, Y., Wang, G., Yan, W. & Liu, L. The biomass comparison of soil microbial carbon and nitrogen of 3 kinds of forest types in subtropics (in Chinese with English abstract). *Chin. Agric. Sci. Bull.* **28**, 14-19 (2012). 61

Eisenhauer, N. *et al.* Plant diversity effects on soil food webs are stronger than those of elevated CO_2 and N deposition in a long-term grassland experiment. *Proc. Natl Acad. Sci. USA* **110**, 6889-6894 (2013). 16

Eisenhauer, N. *et al.* Plant diversity effects on soil microorganisms support the singular hypothesis. *Ecology* **91**, 485-496 (2010). 35

Eisenhauer, N. *et al.* Root biomass and exudates link plant diversity with soil bacterial and fungal biomass. *Sci. Rep.* **7**, 44641 (2017). 62

Fan, F. L., Zhang, F. S., Qu, Z. & Lu, Y. H. Plant carbon partitioning below ground in the presence of different neighboring species. *Soil Biol. Biochem.* **40**, 2266-2272 (2008). 63

Fan, S. & Yang, N. Comparsion of soil microbiology characteristics in five subtropical ecosystems (in Chinese with English abstract). *J. Trop. Subtrop. Bot.* **24**, 635-641 (2016). 64

Fang, S. Z., Liu, D., Tian, Y., Deng, S. P. & Shang, X. L. Tree species composition influences enzyme activities and microbial biomass in the rhizosphere: a rhizobox approach. *PloS One* **8**, e61461 (2013). 65

Finney, D. M., Buyer, J. S. & Kaye, J. P. Living cover crops have immediate impacts on soil microbial community structure and function. *J. Soil Water Conserv.* **72**, 361-373 (2017). 66

Gong, J. R. *et al.* Effect of irrigation on the soil respiration of constructed grasslands in Inner Mongolia, China. *Plant Soil* **395**, 159-172 (2015). 67

Gunina, A., Smith, A. R., Godbold, D. L., Jones, D. L. & Kuzyakov, Y. Response of soil microbial community to afforestation with pure and mixed species. *Plant Soil* **412**, 357-368 (2017). 68

He, Y. *et al.* Profiling of microbial PLFAs: Implications for interspecific interactions due to intercropping which increase phosphorus uptake in phosphorus limited acidic soils. *Soil Biol. Biochem.* **57**, 625-634 (2013). 69

Hu, B. *et al.* Comparison of nitrogen nutrition and soil carbon status of afforested stands established in degraded soil of the Loess Plateau, China. *For. Ecol. Manage.* **389**, 46-58 (2017). 70

Hu, Y., Zhai, M., Wu, J. & Jia, L. Seasonal dynamics of amount and bio-chemical activity of soil microorganisms in pure and mixed stands of poplar and black locust (in Chinese). *Soils* **34**, 42-46 (2002). 71

Huang, Y. *et al.* Changes in soil quality due to introduction of broad-leaf trees into clear-felled Chinese fir forest in the mid-subtropics of China. *Soil Use Manage.* **20**, 418-425 (2004). 72

Jiang, Y. M., Chen, C. R., Liu, Y. Q. & Xu, Z. H. Soil soluble organic carbon and nitrogen pools under mono- and mixed species forest ecosystems in subtropical China. *J. Soils Sed.* **10**, 1071-1081 (2010). 73

Khlifa, R. *Effets de la diversité des arbres sur le fonctionnement de l'écosystème dans deux plantations de forêts tempérées.* Ph.D. Dissertation, Université Laval Québec Canada, (2016). 74

Kong, C.-H., Wang, M.-L., Wang, P., Ni, H.-W. & Meng, X.-R. Reproduction allocation and potential mechanism of individual allelopathic rice plants in the presence of competing barnyardgrass. *Pest Manage. Sci.* **69**, 142-148 (2012). 75

Ladygina, N. & Hedlund, K. Plant species influence microbial diversity and carbon allocation in the rhizosphere. *Soil Biol. Biochem.* **42**, 162-168 (2010). 76

Lange, M. *et al.* Biotic and abiotic properties mediating plant diversity effects on soil microbial communities in an experimental grassland. *PLoS One* **9**, e96182 (2014). 22

Latati, M. *et al.* Intercropping maize and common bean enhances microbial carbon and nitrogen availability in low phosphorus soil under Mediterranean conditions. *Eur. J. Soil Biol.* **80**, 9-18 (2017). 77

Leloup, J. *et al.* Unravelling the effects of plant species diversity and aboveground litter input on soil bacterial communities. *Geoderma* **317**, 1-7 (2018). 78

Li, Q. *et al.* Biochemical and microbial properties of rhizospheres under maize/peanut intercropping. *J. Intergra. Agric.* **15**, 101-110 (2016). 79

Li, X. *et al.* Effects of plant community composition on microbial community in constructed wetlands (in Chinese with English abstract). *Chin. J. Ecol.* **33**, 1508-1514 (2014). 80

Liu, J. *et al.* Effects of tree species and soil properties on the composition and diversity of the soil bacterial community following afforestation. *For. Ecol. Manage.* **427**, 342-349 (2018). 81

Liu, M., Hu, F., He, Y. & Li, H. Seasonal dynamics of soil microbial biomass and its significance to indicate soil quality under different vegetations restored on degraded red soils (in Chinese with English abstract). *Acta Pedolog. Sinica* **40**, 937-944 (2003). 82

Liu, Y., Yang, J., Du, T. & Nie, G. Effect of rehabilitated forest on soil microbial characteristics of severely degraded red soil region (in Chinese with English abstract). *J. Fujian Colleg. For.* **23**, 65-69 (2003). 83

Liuzhuo, M., Liu, Z., Feng, S. & Duan, E. Soil biological characteristics in different kinds of artificial forests in the semi-humid zones of loess plateau (in Chinese with English abstract). *J. Northwest For. Univ.* **24**, 26-31 (2009). 84

Luo, D. *et al.* Impacts of nitrogen-fixing and non-nitrogen-fixing tree species on soil respiration and microbial community composition during forest management in subtropical China. *Ecol. Res.* **31**, 683-693 (2016). 85

Luo, D., Shi, Z., Tang, J., Liu, S. & Lu, L. Soil microbial community structure of monoculture and mixed plantation stands of native tree species in south subtropical China (in Chinese with English abstract). *Chin. J Appl. Ecol.* **25**, 2543-2550 (2014). 86

Malchair, S. *et al.* Do climate warming and plant species richness affect potential nitrification, basal respiration and ammonia-oxidizing bacteria in experimental grasslands? *Soil Biol. Biochem.* **42**, 1944-1951 (2010). 87

Oelbermann, M. & Echarte, L. Evaluating soil carbon and nitrogen dynamics in recently established maize-soyabean inter-cropping systems. *Eur. J. Soil Sci.* **62**, 35-41 (2011). 88

Oelbermann, M., Regehr, A. & Echarte, L. Changes in soil characteristics after six seasons of cereal–legume intercropping in the Southern Pampa. *Geoderma Region.* **4**, 100-107 (2015). 89

Orwin, K. H. & Wardle, D. A. Plant species composition effects on belowground properties and the resistance and resilience of the soil microflora to a drying disturbance. *Plant Soil* **278**, 205-221 (2005). 90

Pan, H. *Soil carbon and nitrogen transformation process research of relationship between soilo microbial community structure of Michelia macclurei, Pinus massoniana plantation (in Chinese with English abstract).* Master's thesis, Guangxi University, (2015). 91

Pausch, J., Zhu, B., Kuzyakov, Y. & Cheng, W. Plant inter-species effects on rhizosphere priming of soil organic matter decomposition. *Soil Biol. Biochem.* **57**, 91-99 (2013). 92

Pereira, E. L., Santos, S. A. P., Arrobas, M. & Patricio, M. S. Microbial biomass and N mineralization in 93

mixed plantations of broadleaves and nitrogen-fixing species. *For. Syst.* **20**, 516-524 (2011).
Porazinska, D. L. *et al.* Relationships at the aboveground-belowground interface: plants, soil biota, and soil processes. *Ecol. Monogr.* **73**, 377-395 (2003). 94
Qu, J. *Effects of intercropping oat and common vetch on forage yield and quality and soil characteristics (in Chinese with English abstract).* Master's thesis, Inner Mongolia Agricultural University, (2017). 95
Rachid, C. T. C. C. *et al.* Mixed plantations can promote microbial integration and soil nitrate increases with changes in the N cycling genes. *Soil Biol. Biochem.* **66**, 146-153 (2013). 96
Rivest, D., Paquette, A., Shipley, B., Reich, P. B. & Messier, C. Tree communities rapidly alter soil microbial resistance and resilience to drought. *Funct. Ecol.* **29**, 570-578 (2015). 97
Salamon, J. A. & Alphei, J. The collembola community of a central European forest: influence of tree species composition. *Eur. J. Soil Biol.* **45**, 199-206 (2009). 98
Sanaullah, M., Blagodatskaya, E., Chabbi, A., Rumpel, C. & Kuzyakov, Y. Drought effects on microbial biomass and enzyme activities in the rhizosphere of grasses depend on plant community composition. *Appl. Soil Ecol.* **48**, 38-44 (2011). 99
Scalise, A. *et al.* Legume-barley intercropping stimulates soil N supply and crop yield in the succeeding durum wheat in a rotation under rainfed conditions. *Soil Biol. Biochem.* **89**, 150-161 (2015). 101
Scalise, A., Pappa, V. A., Gelsomino, A. & Rees, R. M. Pea cultivar and wheat residues affect carbon/nitrogen dynamics in pea-triticale intercropping: A microcosms approach. *Sci. Total Environ.* **592**, 436-450 (2017). 100
Scheu, S. *et al.* The soil fauna community in pure and mixed stands of beech and spruce of different age: trophic structure and structuring forces. *Oikos* **101**, 225-238 (2003). 102
Sharma, R. C. & Banik, P. Baby corn-legumes intercropping systems: I. Yields, resource utilization efficiency, and soil health. *Agroecol. Sust. Food Syst.* **39**, 41-61 (2015). 103
Song, Y. N. *et al.* Effect of intercropping on crop yield and chemical and microbiological properties in rhizosphere of wheat (*Triticum aestivum* L.), maize (*Zea mays* L.), and faba bean (*Vicia faba* L.). *Biol. Fertility Soils* **43**, 565-574 (2007). 104
Sørensen, L. I., Mikola, J. & Kytöviita, M.-M. Defoliation effects on plant and soil properties in an experimental low arctic grassland community – the role of plant community structure. *Soil Biol. Biochem.* **40**, 2596-2604 (2008). 105
Souza, M. F. P., da Silva, M. P., Arf, O. & Cassiolato, A. M. R. Chemical and biological properties of phosphorus-fertilized soil under legume and grass cover (Cerrado region, Brazil). *Rev. Bras. Ciênc. Solo* **37**, 1492-1501 (2013). 106
Spehn, E. M., Joshi, J., Schmid, B., Alphei, J. & Körner, C. Plant diversity effects on soil heterotrophic 107

activity in experimental grassland ecosystems. *Plant Soil* **224**, 217-230 (2000).
Spohn, M. & Chodak, M. Microbial respiration per unit biomass increases with carbon-to-nutrient ratios in forest soils. *Soil Biol. Biochem.* **81**, 128-133 (2015). 108
Steinauer, K. *et al.* Plant diversity effects on soil microbial functions and enzymes are stronger than warming in a grassland experiment. *Ecology* **96**, 99-112 (2015). 109
Steinauer, K., Chatzinotas, A. & Eisenhauer, N. Root exudate cocktails: the link between plant diversity and soil microorganisms? *Ecol. Evol.* **6**, 7387-7396 (2016). 20
Strecker, T., Macé, O. G., Scheu, S. & Eisenhauer, N. Functional composition of plant communities determines the spatial and temporal stability of soil microbial properties in a long-term plant diversity experiment. *Oikos* **125**, 1743-1754 (2016). 29
Su, X., Li, Y., Yang, B. & Li, Q. Effect of plant diversity on soil microbial community in the subtropical forest soil (in Chinese with English abstract). *Chin. J. Ecol.* **37**, 2254-2261 (2018). 110
Sun, C. *Response of rhizospheric microbiology to plant competition and moisture stress (in Chinese with English abstract).* Ph.D. dissertation, Northwest A & F University, (2017). 111
Sun, M. M. *et al.* In situ phytoremediation of PAH-contaminated soil by intercropping alfalfa (*Medicago sativa* L.) with tall fescue (*Festuca arundinacea* Schreb.) and associated soil microbial activity. *J. Soils Sed.* **11**, 980-989 (2011). 112
Sun, Y. M. *et al.* Influence of intercropping and intercropping plus rhizobial inoculation on microbial activity and community composition in rhizosphere of alfalfa (*Medicago sativa* L.) and Siberian wild rye (*Elymus sibiricus* L.). *FEMS Microbiol. Ecol.* **70**, 218-226 (2009). 113
Tan, G. *et al.* Content and seasonal change of soil labile organic carbon under four different plantations in degraded red soil region (in Chinese with English abstract). *Acta Agric. Univ. Jiangxi (Natural Sciences Edition)* **36**, 434-440 (2014). 114
Tang, X. *Effects of water and nitrogen coupling on pasture establishment in HulunBuir (in Chinese with English abstract).* Master's thesis, Chinese Academy of Agricultural Sciences, (2018). 115
Tang, X. Y. *et al.* Increase in microbial biomass and phosphorus availability in the rhizosphere of intercropped cereal and legumes under field conditions. *Soil Biol. Biochem.* **75**, 86-93 (2014). 116
Tang, X. Y. *et al.* Phosphorus availability and microbial community in the rhizosphere of intercropped cereal and legume along a P-fertilizer gradient. *Plant Soil* **407**, 119-134 (2016). 117
Tang, Y., Xu, Y., Zheng, Y. & Lei, B. Effects of wheat and faba bean intercropping on microorganism involved in nitrogen transformation in the rhizosphere soils (in Chinese with English abstract). *J. Agric. Resour. Environ.* **33**, 482-490 (2016). 118
Thakur, M. P. & Eisenhauer, N. Plant community composition determines the strength of top-down control in 119

a soil food web motif. *Sci. Rep.* **5**, 9134 (2015).
Tortorella, D. *et al.* Chemical and biological responses in a Mediterranean sandy clay loam soil under grain legume-barley intercropping. *Agrochimica* **57**, 1-21 (2013). 120
van Eekeren, N. *et al.* A mixture of grass and clover combines the positive effects of both plant species on selected soil biota. *Appl. Soil Ecol.* **42**, 254-263 (2009). 121
Vogel, A., Eisenhauer, N., Weigelt, A. & Scherer-Lorenzen, M. Plant diversity does not buffer drought effects on early-stage litter mass loss rates and microbial properties. *Global Change Biol.* **19**, 2795-2803 (2013). 122
Wagner, D., Eisenhauer, N. & Cesarz, S. Plant species richness does not attenuate responses of soil microbial and nematode communities to a flood event. *Soil Biol. Biochem.* **89**, 135-149 (2015). 123
Waldrop, M. P., Zak, D. R., Blackwood, C. B., Curtis, C. D. & Tilman, D. Resource availability controls fungal diversity across a plant diversity gradient. *Ecol. Lett.* **9**, 1127-1135 (2006). 124
Wang, D. *The interspecific competitive and synergistic effects in the process of Chromolaena odorata invasion (in Chinese with English abstract).* Master's thesis, South China Agricultural University, (2016). 126
Wang, D. *The research on the interspecies allelopathic effect of Juglans manchurian and Larix gmelinii forest plantation (in Chinese with English abstract).* Master's thesis, Northeast Forestry University, (2014). 125
Wang, H. *et al.* Mixed-species plantation with *Pinus massoniana* and *Castanopsis hystrix* accelerates C loss in recalcitrant coniferous litter but slows C loss in labile broadleaf litter in southern China. *For. Ecol. Manage.* **422**, 207-213 (2018). 127
Wang, X., Ge, Y. & Wang, J. Positive effects of plant diversity on soil microbial biomass and activity are associated with more root biomass production. *J. Plant Interact.* **12**, 533-541 (2017). 128
Wardle, D. A. & Nicholson, K. S. Synergistic effects of grassland plant species on soil microbial biomass and activity: Implications for ecosystem-level effects of enriched plant diversity. *Funct. Ecol.* **10**, 410-416 (1996). 130
Wardle, D. A., Bonner, K. I. & Barker, G. M. Stability of ecosystem properties in response to above-ground functional group richness and composition. *Oikos* **89**, 11-23 (2000). 129
Wardle, D. A., Yeates, G. W., Williamson, W. & Bonner, K. I. The response of a three trophic level soil food web to the identity and diversity of plant species and functional groups. *Oikos* **102**, 45-56 (2003). 131
Wu, P. & Xue, J. Effects of three different plantations on soil physicochemical and microbial characteristics in Krast region (in Chinese with English abstract). *J. Nanjing For. Univ. (Natural Sciences Edition)* **39**, 67-72 (2015). 132
Xia, Z.-C., Kong, C.-H., Chen, L.-C., Wang, P. & Wang, S.-L. A broadleaf species enhances an autotoxic conifers growth through belowground chemical interactions. *Ecology* **97**, 2283-2292 (2016). 133

Yan, M. *Effects of different cropping patterns of soybean and maize seedlings on root zone soil carbon and nitrogen mineralization (in Chinese with English abstract).* Master's thesis, Northeast Agricultural University, (2013). 134

Yuan, H. *The research on the interspecies effect of Fraxinus mandshurica and Larix gmelinii forest plantation (in Chinese with English abstract).* Master's thesis, Northeast Forstry University, (2015). 135

Zak, D. R., Holmes, W. E., White, D. C., Peacock, A. D. & Tilman, D. Plant diversity, soil microbial communities, and ecosystem function: are there any links? *Ecology* **84**, 2042-2050 (2003). 13

Zhang, J., Wang, S., Wang, Q. & Liu, Y. Content and seasonal change in soil labile organic carbon under different forest covers (in Chinese with English abstract). *Chin. J. Eco-Agric.* **17**, 41-47 (2009). 136

Zhang, S., Yu, M., Zhu, L. & Qin, X. A study on microbial biomass C, N characteristics in different rehabilitating forests on degraded red soil (in Chinese with English abstract). *Acta Agric. Univ. Jiangxi (Natural Sciences Edition)* **32**, 101-107 (2010). 137

Zhao, J., Zeng, Z. X., He, X. Y., Chen, H. S. & Wang, K. L. Effects of monoculture and mixed culture of grass and legume forage species on soil microbial community structure under different levels of nitrogen fertilization. *Eur. J. Soil Biol.* **68**, 61-68 (2015). 138

Zhao, R. *et al.* Effects of plantation restoration approaches on soil enzyme activities and microbial properties in hilly red soil region (in Chinese with English abstract). *Soils* **44**, 576-580 (2012). 139

Zhou, J. *et al.* Effect of *Cunninghamia lanceolata-Betula luminifera* mixed forests on soil microbial biomass and enzyme activity (in Chinese with English abstract). *J. Northeast For. Univ.* **43**, 83-86 (2015). 140

45

Supplementary Table 2. The Akaike information criterion (AIC) values for the full model (Equation (3) in Methods) and the most parsimonious model, and the effects (*P* value) of intercept (testing whether it differs from zero), the species richness in mixtures (R) and stand age (A) of the most parsimonious models. For all microbial attributes, ecosystem type-associated terms (Equation (3) in Methods) were excluded in the most parsimonious models. The corresponding coefficients and their bootstrapped 95% confidence intervals are presented in Fig. 3. P values are in bold when < 0.05.

Attribute	Full model	The most parsimonious model				
	AIC	AIC	Intercept	ln(R)	A	ln(R) × A
Microbial biomass	-21.2	-32.5	**< 0.001**	**< 0.001**	**< 0.001**	**< 0.001**
Bacterial biomass	-4.2	-34.7	0.054	**0.004**	0.293	**–**
Fungal biomass	87.1	64.9	**0.004**	**0.008**	0.219	**–**
Fungi : bacteria	6.4	-10.7	**0.036**	0.992	**0.046**	**–**
G+ : G-	30.5	1.6	0.900	**0.040**	0.600	**–**
Microbial C : N	124.3	111.1	0.757	0.387	0.265	**–**
Microbial respiration	-75.3	-91.4	**< 0.001**	**< 0.001**	**< 0.001**	**< 0.001**
Metabolic quotient	52.3	43.0	0.300	0.652	0.103	**–**

See Methods for the determination of the most parsimonious model.

Supplementary Table 3. Akaike information criterion (AIC) values among four alternative scenarios with the linear and log-linear species richness (*R*) and stand age (*A*).

Attribute	I		II		III		IV	
	Terms	AIC	Terms	AIC	Terms	AIC	Terms	AIC
Microbial biomass	$R + A + R \times A$	-19.9	$\ln(R) + A + \ln(R) \times A$	**-32.5**	$\ln(R) + \ln(A) + \ln(R) \times \ln(A)$	-21.9	$R + \ln(A) + R \times \ln(A)$	-10.2
Bacterial biomass	$R + A$	-31	$\ln(R) + A$	**-34.7**	$\ln(R) + \ln(A)$	-34.5	$R + \ln(A)$	-31
Fungal biomass	$R + A$	69.5	$\ln(R) + A$	**64.9**	$\ln(R) + \ln(A)$	66.3	$R + \ln(A)$	71.4
Fungi : bacteria	$R + A$	**-11.1**	$\ln(R) + A$	-10.7	$\ln(R) + \ln(A)$	-6.9	$R + \ln(A)$	-7.2
G+: G-	$R + A$	4.3	$\ln(R) + A$	1.6	$\ln(R) + \ln(A)$	**1.5**	$R + \ln(A)$	4.2
Microbial C : N	$R + A$	111.3	$\ln(R) + A$	**111.1**	$\ln(R) + \ln(A)$	112.7	$R + \ln(A)$	112.9
Microbial respiration	$R + A + R \times A$	-77.5	$\ln(R) + A + \ln(R) \times A$	-91.4	$\ln(R) + \ln(A) + \ln(R) \times \ln(A)$	**-92.6**	$R + \ln(A) + R \times \ln(A)$	-79.1
Metabolic quotient	$R + A$	43.0	$\ln(R) + A$	**43.0**	$\ln(R) + \ln(A)$	45.4	$R + \ln(A)$	45.3

Supplementary Table 4. The effect (*P* values) of the technical method on natural log response ratios (ln*RR*) of studied microbial biomass.

Attribute	df	*P*
Microbial biomass	2, 76	0.212
Bacterial biomass	1, 38	0.555
Fungal biomass	1, 117	0.524
Bacteria : Fungi	1, 72	0.478

Linear mixed effect models used Satterthwaite approximation for degrees of freedom (df).

Supplementary Table 5. The effect (*P* values) of soil depth on natural log response ratios (ln*RR*) of studied microbial attributes.

Attribute	df	*P*
Microbial biomass	1, 367	0.557
Bacterial biomass	1, 36	0.463
Fungal biomass	1, 33	0.183
Fungi : bacteria	1, 33	0.210
G+: G-	1, 6	0.729
Microbial C : N	1, 77	0.273
Microbial respiration	1, 77	0.203
Metabolic quotient	1, 55	0.162

Linear mixed effect models used Satterthwaite approximation for degrees of freedom (df)

Supplementary Methods

The interpretation of the effects of reduced plant diversity on microbial biomass and respiration over time.

The ln*RR* when the plant richness in mixtures was R_1 for a period of A:

$$\ln RR_1 = \beta_0 + \beta_1 \cdot \ln(R_1) + \beta_2 \cdot A + \beta_3 \cdot \ln(R_1) \times A + \pi_{study} + \varepsilon \tag{1}$$

The ln*RR* when plant richness in mixtures was R_α (α % lower species richness than R_1) for a period of A:

$$\ln RR_\alpha = \beta_0 + \beta_1 \cdot \ln(R_\alpha) + \beta_2 \cdot A + \beta_3 \cdot \ln(R_\alpha) \times A + \pi_{study} + \varepsilon \tag{2}$$

Supplementary Equation 2 - Supplementary Equation 1:

$$\ln RR_\alpha - \ln RR_1 = (\beta_1 + \beta_3 \cdot A) \times (\ln(R_\alpha) - \ln(R_1)) \tag{3}$$

$$\ln(RR_\alpha / RR_1) = \ln((R_\alpha / R_1)^{\beta_1 + \beta_3 \cdot A}) \tag{4}$$

$$(X_{t\_\alpha} / X_{c\_\alpha}) / (X_{t\_1} / X_{c\_1}) = RR_\alpha / RR_1 = (R_\alpha / R_1)^{\beta_1 + \beta_3 \cdot A} \tag{5}$$

We assumed that the mean value of monocultures (X_c) did not vary with the number of monocultures of different species, i.e., X_{c_α} was no different from X_{c_1}:

$$X_{t\_\alpha} / X_{t\_1} = P_\alpha = (R_\alpha / R_1)^{\beta_1 + \beta_3 \cdot A} \tag{6}$$

where model terms were described in Equation (3) in the main text.
