# Supplementary material for: Meta-analysis shows positive effects of plant diversity on microbial biomass and respiration
Source: Nat Commun. 2019 Mar 22;10:1332. doi: 10.1038/s41467-019-09258-y (PMC6430801; doi:10.1038/s41467-019-09258-y)
Supplement: Supplementary file 3 — Description of Additional Supplementary Files [file 41467_2019_9258_MOESM3_ESM.pdf]

Description of Additional Supplementary Files

File Name: Supplementary Software 1
Description: R scripts needed to reproduce the statistical analysis and produce Figs. 1–5 and Supplementary Figs. 1–2 are all included
