# Supplementary material for: Meta-analysis shows positive effects of plant diversity on microbial biomass and respiration
Source: Nat Commun. 2019 Mar 22;10:1332. doi: 10.1038/s41467-019-09258-y (PMC6430801; doi:10.1038/s41467-019-09258-y)
Supplement: Supplementary file 6 — Source Data [file 41467_2019_9258_MOESM6_ESM.pdf]

nature research

Corresponding author(s): Dr. Han Y. H. Chen

Last updated by author(s): Feb 18, 2019

Reporting Summary

Nature Research wishes to improve the reproducibility of the work that we publish. This form provides structure for consistency and transparency in reporting. For further information on Nature Research policies, see Authors & Referees and the Editorial Policy Checklist.

Statistics

For all statistical analyses, confirm that the following items are present in the figure legend, table legend, main text, or Methods section.

n/a	Confirmed	
☐	☒	The exact sample size (*n*) for each experimental group/condition, given as a discrete number and unit of measurement
☐	☒	A statement on whether measurements were taken from distinct samples or whether the same sample was measured repeatedly
☐	☒	The statistical test(s) used AND whether they are one- or two-sided<br>*Only common tests should be described solely by name; describe more complex techniques in the Methods section.*
☐	☒	A description of all covariates tested
☐	☒	A description of any assumptions or corrections, such as tests of normality and adjustment for multiple comparisons
☐	☒	A full description of the statistical parameters including central tendency (e.g. means) or other basic estimates (e.g. regression coefficient) AND variation (e.g. standard deviation) or associated estimates of uncertainty (e.g. confidence intervals)
☐	☒	For null hypothesis testing, the test statistic (e.g. F, t, r) with confidence intervals, effect sizes, degrees of freedom and P value noted<br>*Give P values as exact values whenever suitable.*
☒	☐	For Bayesian analysis, information on the choice of priors and Markov chain Monte Carlo settings
☐	☒	For hierarchical and complex designs, identification of the appropriate level for tests and full reporting of outcomes
☐	☒	Estimates of effect sizes (e.g. Cohen's *d*, Pearson's *r*), indicating how they were calculated

Our web collection on statistics for biologists contains articles on many of the points above.

Software and code

Policy information about availability of computer code

Data collection	Plot Digitizer version 2.0 (Department of Physics at the University of South Alabama, Mobile, AL, USA)
Data analysis	The data analysis was conducted in R 3.5.1 (R Core Team, 2018), using packages including 'data.table (version 1.11.4)', 'lme4 (version 1.1-17)', 'lmerTest (version 3.0-1)', 'ggplot2 (version 2.2.1)', 'maps (version 3.2.0)' and 'cowplot (version 0.9.2)'.

For manuscripts utilizing custom algorithms or software that are central to the research but not yet described in published literature, software must be made available to editors/reviewers. We strongly encourage code deposition in a community repository (e.g. GitHub). See the Nature Research guidelines for submitting code & software for further information.

Data

Policy information about availability of data

All manuscripts must include a data availability statement. This statement should provide the following information, where applicable:

- Accession codes, unique identifiers, or web links for publicly available datasets
- A list of figures that have associated raw data
- A description of any restrictions on data availability

The authors declare that the data and R script supporting the findings of this study will be available within its supplementary information files when publishing. The source data underlying Figs. 1–5 and Supplementary Figs. S1–S2 are provided as a Source Data file.

Field-specific reporting

Please select the one below that is the best fit for your research. If you are not sure, read the appropriate sections before making your selection.

☐ Life sciences ☐ Behavioural & social sciences ☒ Ecological, evolutionary & environmental sciences

For a reference copy of the document with all sections, see nature.com/documents/nr-reporting-summary-flat.pdf

Ecological, evolutionary & environmental sciences study design

All studies must disclose on these points even when the disclosure is negative.

Study description	We conducted a global meta-analysis with 1332 paired observations of plant monocultures and mixtures from 106 studies, to evaluate the responses of soil microbial biomass, composition, and respiration to plant species mixtures. We investigated whether the plant mixture effects on the microbial attributes were affected by plant species richness, stand age, ecosystem types, climatic factors and soil depth.
Research sample	The data for this study was obtained from 106 peer-reviewed publications that investigated the effects of plant diversity on microbial biomass, composition, and respiration, using the Web of Science and Google Scholar, up to 1st November 2018. The 106 publications were listed in Appendix 2 in Supplementary Materials.
Sampling strategy	We searched all peer-reviewed publications that investigated the effects of plant diversity on microbial biomass, composition, and respiration, using the Web of Science and Google Scholar, up to 1st November 2018. Various keyword combinations were employed for the search, such as "(species diversity OR tree diversity OR richness OR mix species OR mix tree OR mixture OR intercrop) AND (microbial abundance OR microbial biomass OR microbe OR microbial OR soil biota OR microbial community OR fungi OR bacteria OR microbial biomass nitrogen OR microbial respiration OR basal respiration OR microbial activity)". We reviewed each article to determine whether the studies met the following criteria: 1) isolated the effects of plant diversity from other factors, such as stand age and topography; 2) the microbial attributes could be extracted directly from the text, tables, and figures.<br>The sample size of each treatment (plant mixture) and control (plant monoculture) was directly recorded from the original publication to estimate the weight for each observation.
Data collection	Chen Chen and Xinli Chen collected the data. For each study, we extracted microbial attributes including microbial biomass, bacterial biomass, fungal biomass, fungi:bacteria ratio, gram-positive bacteria to gram-negative bacteria ratio, microbial carbon:nitogen ratio, microbial respiration and metabolic quotient. We used Plot Digitizer version 2.0 (Department of Physics at the University of South Alabama, Mobile, AL, USA) to digitally extract data from figures when the results were graphically reported. We obtained plant species richness, species identity, geographical location (latitude, longitude, and altitude), experimental system type, stand age, soil sampling depth, and mean annual temperature (MAT) from original publications. The stand age referred to the years between stand establishment or experiment initiation, and the measurement of soil microbial attributes. For those studies conducted in natural climatic condition, including forests and grasslands, we derived an aridity index (calculated as mean annual precipitation divided by mean annual potential evapotranspiration, where a higher aridity index indicated lower aridity) from a Global Aridity and PET Database (Zomer et al., 2008). When the MAT was not available from original publications, it was derived based on geographic locations using the WorldClim version 2 dataset (Fick et al., 2017).<br>When different publications included the same data from one study, we recorded the data only once. When a study included plant mixtures of different species combinations, we considered them as distinct observations. Also, when a publication included several experiments under different abiotic conditions, such as different locations, treatments, stand ages, and soil layers, we considered them as different observations. We obtained a meta-dataset of 1332 observations from 106 studies that involved the mixing of live plants in natural forests, planted forests, planted grasslands, croplands, and containers.
Timing and spatial scale	The searching for publication and data collection continuously proceeded from August 15th, 2017 to November 1st, 2018.<br>The timing scale of the observations in original studies ranged from 1992 to 2016.<br>The observations were distributed in global terrestrial ecosystems.
Data exclusions	No data were excluded from the analyses.
Reproducibility	All attempts to repeat the analysis and results were successful.
Randomization	Bootstrapping sampling was used.
Blinding	Complete blinding in bootstrapping process.

Did the study involve field work? ☐ Yes ☒ No

Reporting for specific materials, systems and methods

We require information from authors about some types of materials, experimental systems and methods used in many studies. Here, indicate whether each material, system or method listed is relevant to your study. If you are not sure if a list item applies to your research, read the appropriate section before selecting a response.

Materials & experimental systems

n/a	Involved in the study
☒	☐ Antibodies
☒	☐ Eukaryotic cell lines
☒	☐ Palaeontology
☒	☐ Animals and other organisms
☒	☐ Human research participants
☒	☐ Clinical data

Methods

n/a	Involved in the study
☒	☐ ChIP-seq
☒	☐ Flow cytometry
☒	☐ MRI-based neuroimaging
